# De Novo Transcriptomic Analyses Revealed Some Detoxification Genes and Related Pathways Responsive to Noposion Yihaogong^®^ 5% EC (Lambda-Cyhalothrin 5%) Exposure in *Spodoptera frugiperda* Third-Instar Larvae

**DOI:** 10.3390/insects12020132

**Published:** 2021-02-03

**Authors:** Muhammad Hafeez, Xiaowei Li, Zhijun Zhang, Jun Huang, Likun Wang, Jinming Zhang, Sakhawat Shah, Muhammad Musa Khan, Fei Xu, G. Mandela Fernández-Grandon, Myron P. Zalucki, Yaobin Lu

**Affiliations:** 1 State Key Laboratory for Managing Biotic and Chemical Threats to the Quality and Safety of Agro-products, Institute of Plant Protection and Microbiology, Zhejiang Academy of Agricultural Sciences, Hangzhou 310021, China; hafeez_203@yahoo.com (M.H.); lixiaowei1005@163.com (X.L.); zhijunzhanglw@hotmail.com (Z.Z.); junhuang1981@aliyun.com (J.H.); wanglikun1314@sina.cn (L.W.); zhanginsect@163.com (J.Z.); 2 Hubei Insect Resources Utilization and Sustainable Pest Management Key Laboratory, College of Plant Science and Technology, Huazhong Agricultural University, Wuhan 430070, China; shahentomology@webmail.hzau.edu.cn; 3 Key Laboratory of Bio-Pesticide Innovation and Application, South China Agricultural University, Guangzhou 510642, China; drmusakhan@outlook.com; 4 Central Laboratory of Zhejiang Academy of Agricultural Sciences, Hangzhou 310021, China; fxu@zaas.ac.cn; 5 Natural Resources Institute, University of Greenwich, Chatham Maritime, Kent ME4 4TB, UK; m.fernandez-grandon@greenwich.ac.uk; 6 School of Biological Sciences, The University of Queensland, Brisbane, QLD 4072, Australia; m.zalucki@uq.edu.au

**Keywords:** transcriptome analysis, *S. frugiperda*, Noposion Yihaogong^®^ 5% EC, lambda-cyhalothrin, detoxification genes, pathways

## Abstract

**Simple Summary:**

Insect pest resistance to synthetic insecticides is a major problem that limits efficient management and thus decreases productivity for farmers and increases the use of harmful materials that pollute the environment and endanger humans and beneficial organisms. A major approach for resistance management is understanding how insect pest field populations develop resistance at molecular levels. To provide a comprehensive insight into the resistance mechanisms of *Spodoptera frugiperda* larvae to lambda-cyhalothrin 5%, we investigated the molecular basis of resistance mechanism in field collected population of fall armyworm (*Spodoptera frugiperda*) to lambda-cyhalothrin 5% insecticide, a pyrethroid insecticide by using de novo transcriptomics analysis. We found that resistance to lambda-cyhalothrin 5% can be metabolic by increasing the levels of detoxifying enzymes such as P450, GST and UGT and related genes to insecticide resistance in the field population. The obtained transcriptome information provides large gene resources available for further studying the resistance development of *Spodoptera frugiperda* to pesticides. The DGE data provide comprehensive insights into the gene expression profiles of fall armyworm (*Spodoptera frugiperda*) to lambda-cyhalothrin 5% and will facilitate the study of the role of each gene in lambda-cyhalothrin resistance development.

**Abstract:**

The fall armyworm, *Spodoptera frugiperda* (J.E. Smith) (Lepidoptera: Noctuidae), is a polyphagous, invasive insect pest which causes significant losses in important crops wherever it has spread. The use of pesticides in agriculture is a key tool in the management of many important crop pests, including *S. frugiperda*, but continued use of insecticides has selected for various types of resistance, including enzyme systems that provide enhanced mechanisms of detoxification. In the present study, we analyzed the de novo transcriptome of *S. frugiperda* larvae exposed to Noposion Yihaogong^®^ 5% emulsifiable concentrate (EC) insecticide focusing on detoxification genes and related pathways. Results showed that a total of 1819 differentially expressed genes (DEGs) were identified in larvae after being treated with Noposion Yihaogong^®^ 5% EC insecticide, of which 863 were up- and 956 down-regulated. Majority of these differentially expressed genes were identified in numerous Kyoto Encyclopedia of Genes and Genomes (KEGG) pathways, including metabolism of xenobiotics and drug metabolism. Furthermore, many of *S. frugiperda* genes involved in detoxification pathways influenced by lambda-cyhalothrin stress support their predicted role by further co-expression network analysis. Our RT-qPCR results were consistent with the DEG’s data of transcriptome analysis. The comprehensive transcriptome sequence resource attained through this study enriches the genomic platform of *S. frugiperda*, and the identified DEGs may enable greater molecular underpinnings behind the insecticide-resistance mechanism caused by lambda-cyhalothrin.

## 1. Introduction

The fall armyworm, *Spodoptera frugiperda* (J.E. Smith) (Lepidoptera: Noctuidae), is an important insect pest of major crops occurring mainly in the tropical and subtropical regions of the American continent [1,2]. In recent years, it has invaded West and Central Africa, where it has been responsible for substantial crop losses and threatens food security in the region [3]. *S. frugiperda* has continued to spread globally and has recently been found in India [4] and southwest China (southwest Yunnan province), where, in January 2019, larvae were found in corn [4,5,6]. The larvae of this insect are highly polyphagous, feeding on a diverse range of host plant species (more than 80 plant species) [7]. Among its host plants are economically important food crops such as maize, rice, sorghum [8], and infrequently, cotton crop [9]. Maize is the preferred host of *S. frugiperda* with yield losses of between 15% and 73% during an outbreak [9,10,11]. China is the world’s second-largest producer of maize, and the crop is planted over large areas in all provinces, and maize serves as food, feed, and industrial material [12,13].

For decades, synthetic insecticides have been used as the main tool for the control of *S. frugiperda,* but increased insecticide resistance hinders our ability to control this destructive insect in the field, and thus threatens agricultural crop production wherever the species is now found [14,15,16]. Nevertheless, increased spread and incidence of *S. frugiperda* has led to increasing and intensive applications of synthetic pesticides to mitigate losses [17]. However, the long-term and widespread use of pesticides has resulted in significant damage to the environment as well as the development of high levels of resistance in insect pest populations [18,19,20,21,22]. The first report of insecticide resistance in *S. frugiperda* was to the carbamate insecticide, carbaryl [23]. Since then, high levels of resistance to pyrethroid, organophosphate, and diamide insecticides has been documented in field populations from different countries [14,18,21,22,24], and even laboratory-selected populations of *S. frugiperda* showed significant resistance ratios of more than 40-fold to lambda-cyhalothrin, a pyrethroid [18].

Resistance to synthetic insecticides mainly consists of two mechanisms: target-site resistance and metabolic resistance [25]. Metabolic resistance has been identified in a range of insect species with resistance to different insecticides due to the gene amplification, overexpression, and/or modification of gene-encoding members of the detoxification enzymes, like cytochrome P-450s (P-450s), glutathione S-transferases (GSTs), and carboxylesterases (CEs) [26,27]. In a previous study, the biochemical characterization of resistance in *S. frugiperda* to a different group of insecticides suggested that resistance was due to both insensitivity of the target site and detoxification of insecticides by metabolic enzymes [14]. In recent years, the induced expression of certain enzymes’ genes related to resistance in insect pests have been reported. For example, several genes (CYP6B8, and CYP321A1, CYP321A7, and CYP321A9) from the cytochrome P-450 family can be induced to metabolize several allelochemicals and insecticides in *Spodoptera litura* and *Helicoverpa zea* [28,29,30]. Similarly, the transcription level of many CYP450, GST, and UDP-glucuronosyltransferases (UGT) genes are significantly upregulated in *Bombyx mori*, *Spodoptera exigua*, and *S. frugiperda* after exposure to different allelochemicals and insecticides [27,31,32]. 

In recent years, researchers have used new technologies and advances in genomic research to identify mechanisms for identifying and potentially controlling pest insecticide resistance. The development of novel next-generation sequencing (NGS) has brought tremendous progress to genomic research in a large number of non-model organisms. RNA-seq data in insect pests would further serve as a useful resource to study aspects of pest control [33,34,35]. Although insecticide resistance mechanisms have been reported before, further research is needed, particularly on the common resistance mechanisms of pests to different insecticides. In this study, we used the high-throughput Illumina HiSeq4000 (Illumina, San Diego, CA, USA) platform to acquire whole-body de novo transcriptome analysis of *S. frugiperda.* Our focus was to quantify the expression levels of key detoxification genes and related pathways in *S. frugiperda.* This can be used as a resource to provide insights into insecticide-resistance mechanisms following treatment with lambda-cyhalothrin. The data generated in this study provide abundant resources based on directed sequencing that will be useful to our understanding of the molecular insecticide resistance of *S. frugiperda* larvae and provide us with new pathways into pest management.

## 2. Material and Methods

### 2.1. Insect Collection and Rearing

The field populations of the *Spodoptera frugiperda* of different larval stages were collected in August 2019 from two different cornfields (Ping Hu, Zhejiang China). Larvae were reared on an artificial diet [36] in a climate control chamber at 25 ± 2 °C with a 14:10 h light:dark photoperiod in the Institute of Plant protection and Microbiology, Zhejiang Academy of Agricultural Sciences Hangzhou, China, for two generations to obtain a more homogenous population before they were used for experiments. Following pupation, newly emerged adults were sexed with mating pairs placed together in cages and provided with 10% honey solution as a food source. A laboratory-susceptible (not true susceptible) population was collected from a maize research field, Yunnan province, in Feb-2019 and maintained on an artificial diet without exposure to insecticides. The early third-instar larvae of the F2 generation from the Zhejiang field population were used for toxicity bioassays and unselected larvae of the same population of the F2 generation were used as a control treatment.

### 2.2. Insecticide and Toxicity Bioassays for Dose Selection

The commercial insecticide product used in the bioassay according to previously reported methods [16,37] was: lambda-cyhalothrin (Noposion Yihaogong^®^ 5% emulsifiable concentrate (EC) insecticide) Shenzhen Noposion Agrochemical Co. Ltd., Shenzhen, China. Composition: lambda-cyhalothrin: 5%, co-solvent: 8% ethyl acetate, solvent: 72% water, stabilizer: 3% glycerol (antifreeze) and trade secret: 12%. A diet-incorporation method for feeding bioassays was used to calculate the dose-mortality response of the early third-instar *S. frugiperda* larvae based on methodology previously reported by Carvalho et al. [20]. Artificial diet-incorporation assays were conducted in small transparent Petri dishes as described previously by Bolzan et al. [38]. The stock solution of Noposion Yihaogong^®^ 5% EC insecticide was prepared and then six serial dilutions ranging from 4.686 to 150 and 0.156 to 5.00 mg-L^−1^ for field population and for lab populations (each concentration was replicated three times) were mixed thoroughly into the semisynthetic diet before solidification of agar (40–45 °C) [39]. Distilled water in the semisynthetic diet was used as a control. Diet supplemented with serial concentrations of test insecticide was cut into small cubes and placed into the small transparent Petri dishes (5-cm diameter), and three small transparent Petri dishes were used for each concentration, including control treatment. A total of 420 third-instar larvae were then transferred onto the contaminated diet (twenty larva per Petri plate), including control treatment. The toxicity bioassays were performed at 25 ± 2 °C with a 14:10 h light:dark photoperiod. Mortality was calculated after 72 h. Larvae were considered alive if they were able to show movement in a coordinated manner when touched with a small soft brush. Control mortality was less than 10%. Probit analysis was done to calculate the lethal concentrations LC_50_ and LC_25_ values for each assay of insecticide using the POLO-PC software package (LeOra Software, Berkeley, CA, USA) [40].

### 2.3. Samples’ Preparation for RNA-Sequence

The early third-instar healthy larvae of *S. frugiperda* were exposed to a sublethal concentration (LC_25_) of Noposion Yihaogong^®^ 5% EC (15.871 mg-L^−1^) for 72 h. Larvae taken from the same population fed on diet without Noposion Yihaogong^®^ 5% EC insecticide served as a control treatment. After 72 h post-exposure, 36 surviving larvae (whole-body) were collected from the treated and untreated group and transferred to micro-tubes (Eppendorf, Hamburg, Germany) on ice and put into liquid nitrogen for 5 min, then stored at −80 °C until RNA extraction. Total RNA was extracted for transcriptomic analyses from three independent biological replicates (12 larvae in each replicate) for each treatment, including untreated as a control. 

### 2.4. Total RNA Isolation, cDNA Library Construction and Illumina Sequencing

Total RNA from Noposion Yihaogong^®^ 5% EC-treated and unselected groups was extracted using Trizol reagent (Invitrogen, Carlsbad, CA, USA) following the protocol according to manufacturer’s instructions. The total RNA quantity and integrity was analyzed using a Bioanalyzer 2100 device and RNA 1000 Nano Lab Chip Kit (Agilent, Santa Clara, CA, USA) with RIN number > 7.0. Poly (A) RNA was purified from total RNA (5 μg) using poly-T oligo-attached magnetic beads using two rounds of purification. Following purification, the mRNA was fragmented into small pieces using divalent cations under an elevated temperature. The cleaved RNA fragments were reverse-transcribed to create the final library of the cDNA in accordance with the protocol for the RNA-Seq sample preparation kit (Illumina, San Diego, CA, USA). The average insert size for the paired-end libraries was 300 bp (±50 bp). The paired-end sequencing was performed on an Illumina Hiseq4000 at the LC Sciences, Houston, TX, USA. 

### 2.5. De Novo Assembly, Unigene Annotation and Functional Classification

Cutadapt [41] and perl scripts in-house were used to remove the reads that contained adaptor contamination, low-quality bases and undetermined bases. Following this, sequence quality was verified using FastQC (http://www.bioinformatics.babraham.ac.uk/projects/fastqc/), including the Q20, Q30 and GC-content of the clean data. All downstream analyses were based on clean data of high quality. De novo assembly of the transcriptome was performed with Trinity 2.4.0 [42]. Trinity groups transcripts into clusters based on shared sequence content. Transcript clusters of this nature were very loosely referred to as a ‘gene’. The trinity assemblies were further examined using TransDecoder (https://github.com/TransDecoder/TransDecoder) to remove false assembled transcripts. The longest transcript in the cluster was chosen as the ‘gene’ sequence (aka Unigene). All assembled Unigenes were aligned against the non-redundant (Nr) protein database (http://www.ncbi.nlm.nih.gov/), Gene Ontology (GO) (http://www.geneontology.org), Swiss Prot (http://www.expasy.ch/sprot/), Kyoto Encyclopedia of Genes and Genomes (KEGG) (http://www.genome.jp/kegg/) and eggnog (http://eggnogdb.embl.de/) databases using DIAMOND, with a threshold E-value of 1 × 10^−5^ [43]. 

### 2.6. Differentially Expressed Gene (DEGs) Analysis

Salmon [44] was used to perform expression level for Unigenes by calculating Transcripts Per Kilobase Million (TPM) [45]. The differentially expressed Unigenes were selected with log2 (fold change) > 1 or log2 (fold change) < −1 and with statistical significance (*p*-value < 0.05) by R package edgeR [46]. Next, GO and KEGG enrichment analyses were performed on the differentially expressed Unigenes by Perl scripts in-house.

### 2.7. Study of Gene Interaction Network with Relation to Detoxification Pathways

To study the correlation of genes with respect to five detoxification pathways under lambda-cyhalothrin stress, co-expression network analysis was performed using Cytoscape software (version 3.3.0, Cytoscape, San Diego, CA, USA) to construct a co-expression regulation network of the genes and to determine the relationships among them according to the method described previously [46]. The co-expression network map was made with *p n* > 0.95 as the threshold [47]. Furthermore, we identified major detoxification DEGs from five different pathways from transcriptome under lambda-cyhalothrin stress.

### 2.8. The RT-qPCR Validation of Differentially Expressed Candidate Genes

RT-qPCR was performed to compare the relative mRNA abundance of candidate genes. Total RNAs from treated and untreated samples were extracted using Trizol Reagent (Takara, Kyoto, Japan) as described above. TransScript One-Step gDNA Removal and cDNA Synthesis SuperMix (TransGene Biotech.co., Ltd. Haidian District, Beijing, China 100192 were used to synthesize cDNA from 1 μg of total RNA template. The reaction mixture of 20 μL consisted of Anchored Oligo(dT)18 Primer (0.5 μg/μL) 1 μL, 2× TS Reaction Mix 10 μL, TransScript^®^ RT/RI Enzyme Mix 1 μL, gDNA Remover 1 μL and RNase-free Water 5 μL. The primers were designed using Premier 5 software, and the sequences of the primers used for candidate genes are listed in Supplementary Table S1. The RT-qPCR analysis was performed with three biological (12 larvae in each replicate) and three technical replicates in each treatment, including control, by using a CFX96™ Real-Time PCR Detection System (Bio-Rad, Hercules, CA, USA) with SsoFast EvoGreen^®^ SuperMix (BIO RAD, Hercules, CA, USA). The RT-qPCR mixture consisted of 20 μL of total volume containing 10 μL of 2× PCR mixture (Sangdon Biotech, Shanghai, China), 0.5 μL of each sense and antisense primers (10 μM), 3 μL of cDNA template and 6 μL of dd H_2_O. The protocol for qRT-PCR was used as follows: 95 °C for 30 s, followed by 40 cycles at 95 °C for 5 s and at 60 °C for 30 s. GADPH (GenBank: KC262638.1) and the ribosomal protein S30 (NCBI locus AF400225) were used as the internal control gene for normalization. All reactions were run in triplicate and fold changes were calculated using the 2^−ΔΔCT^ method by Livak and Schmittgen [48]. 

### 2.9. Statistical Analysis

Concentration-mortality data was analyzed by Probit analysis [49] with POLO 2.0 program LeOra Software (www.leorasoftware.com) to determine the LC_50_ value, standard errors, slopes and 95% fiducial limit (FL). All data including RT-qPCR were analyzed using the statistical software SPSS (version 19.0, SPSS Inc., Chicago, IL, USA). The statistically significant mean values of the treatments were calculated using analysis of variance (ANOVA), and the important variations among the treatments were determined using the Student’s *t* test (*p* < 0.05).

## 3. Results 

### 3.1. Toxicity of Noposion Yihaogong^®^ 5% EC insecticide

Lethal and sublethal concentrations (LC_50_ and LC_25_) of Noposion Yihaogong^®^ 5% EC treatment of the field-collected population for early third-instar larvae of *S. frugiperda* and the lab-susceptible strain at 72 h were identified (Table 1). The results showed that the toxicity (LC_50_ and LC_25_ values) was estimated to be approximately 40.62 and 15.87 µg-g^−1^ for the field-collected population and for the lab-susceptible population was 1.3 and 0.54 µg-g^−1^ (Table 1). The estimated sublethal concentration (LC_25_) was further applied for de novo transcriptome analysis Noposion Yihaogong^®^ 5% EC treatment compared with unselected as a control from the same field-collected population of Zhejiang province. 

### 3.2. Illumina Sequencing and De Novo Assembly

To investigate the effect of Noposion Yihaogong^®^ 5% EC on the third-instar *S. frugiperda* larvae, Illumina Hiseq4000 technology was used to sequence six cDNA libraries from the whole body of the *S. frugiperda* larvae exposed to a sublethal concentration of Noposion Yihaogong^®^ 5% EC insecticide and for the unselected group. In total, raw reads for Noposion Yihaogong^®^ 5% EC-1, Noposion Yihaogong^®^ 5% EC-2, Noposion Yihaogong^®^ 5% EC-3, unselected-1, unselected-2 and unselected-3 were 55,781,304, 57,178,320, 44,957,636, 53,871,434, 54,235,678 and 56,970,122, respectively. After mapping to the reference genome (https://bipaa.genouest.org/v3.1) and the junction database, a total number of 53,123,060, 54,393,760, 42,904,598, 51,566,052, 51,465,482 and 54,229,496 clean reads were generated with more than 90% validity (Table 2). Altogether, all libraries were good quality with an average percentage of Q20 (97.98%) and Q30 (93.75%) containing an average GC percentage of 50.07%, respectively (Table 2). Furthermore, a total of 66,501 transcripts were assembled from all the transcriptome data and the clean reads were assembled into 26,814 unigenes. The minimum and maximum size of unigenes were 201 and 12,042 bp, with mean size of 516 bp, and GC content and N50 were 40.78 bp and 1333, respectively (Supplementary Table S2).

### 3.3. Functional Annotation of the S. frugiperda-Treated Larvae Unigenes

**Figure 1:**
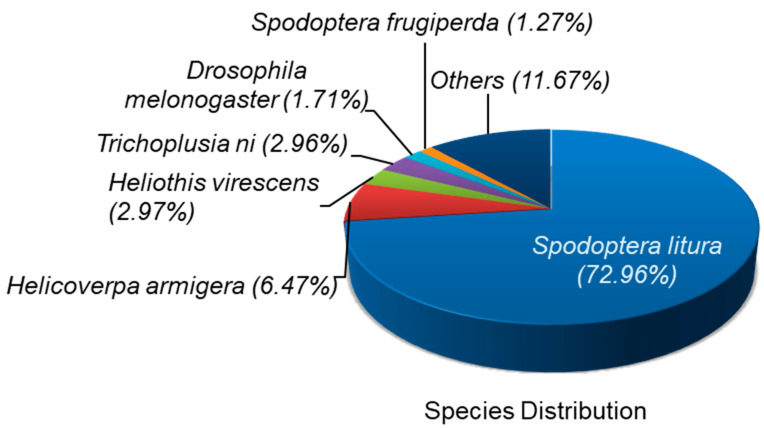
De novo transcriptome analysis of unselected and Noposion Yihaogong^®^ 5% EC insecticide-exposed larvae. Pie chart showing the result from annotation gene ontology annotation and classification of the *Spodoptera frugiperda* transcriptome.

The functional annotation of 26,814 unigene sequences were all aligned against six different public databases, including nr, GO, KEGG, Pfam, Swiss-Prot and eggNOG. The results from functional annotation unigene sequences showed that 13,585 (50.66%), 9115 (33.99%), 9105 (33.96%), 9434 (35.18%), 8055 (30.04%) and 12,465 (46.49%) unigenes matched to nr, GO, KEGG, Pfam, Swiss-Prot and eggNOG protein databases, respectively (Table 3). After nr database annotation, the species distribution of the best-match result for each sequence demonstrated that 73% of the unigenes had highest homology against sequences of *Spodoptera litura*, followed by *Helicoverpa armigera* (6.47%), *Heliothis virescens* (2.97%) *Trichoplusia ni* (2.96%) and *Drosophila melanogaster* (1.71) (Figure 1). 

### 3.4. Differentially Expressed Unigenes in Noposion Yihaogong^®^ 5% EC Insecticide-Treated S. frugiperda Larvae

**Figure 2:**
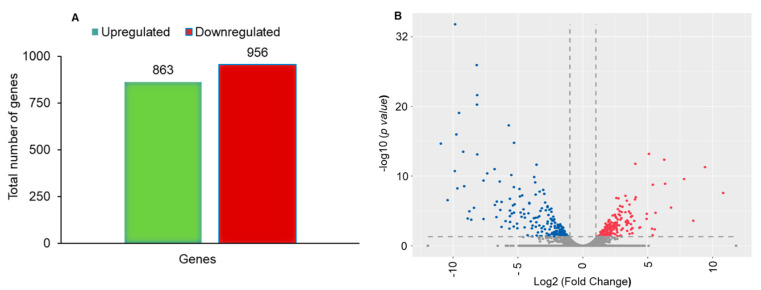
De novo transcriptome analysis of unselected and Noposion Yihaogong^®^ 5% EC insecticide-exposed larvae. (**A**) Bar graph showing total number of up- and down-regulated differentially expressed genes (DEGs). (**B**) Volcano plot analysis of differentially regulated genes in untreated and Noposion Yihaogong^®^ 5% EC insecticide-exposed sample. DeSeq was used for determining upregulation and downregulation of genes based upon relative Fragments Per Kilobase Million (FPKM) counts (*x*-axis: log2fold change; *y*-axis: −log (*p*-value)).

A total of 1819 differentially expressed unigenes (DEGs) were found between Noposion Yihaogong^®^ 5% EC insecticide-treated and the untreated group. Of these, 863 (47.44%) were upregulated and 956 (52.56%) were downregulated (Figure 2A). To ascertain differential gene expression in control as compared to Noposion Yihaogong^®^ 5% EC-exposed samples, a volcano plot was constructed with a log2 fold-change > 1 for upregulated genes and < −1 for downregulated genes (Figure 2B).

### 3.5. Gene Ontology (GO) Enrichment Analysis

**Figure 3:**
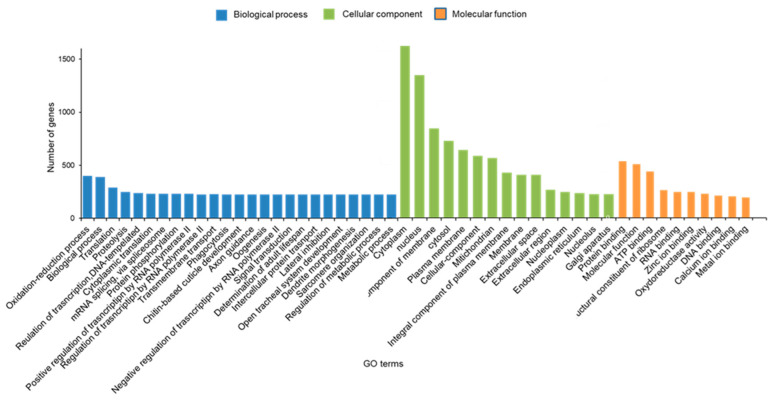
Gene Ontology (GO) enriched terms of differentially expressed genes (DEGs) of *Spodoptera frugiperda* larvae after exposure to a Noposion Yihaogong^®^ 5% EC (TR) versus the control group (unselected). The *x*-axis lists the sub-GO terms under categories of biological process, cellular component and molecular function. The *y*-axis is the number of DEGs involved in each term.

Gene ontology (GO) assignments were used to functionally classify the predicted unigenes from the whole body of *S. frugiperda*. According to the sequence similarity, 4045 unigenes (15.12%) were annotated and classified into 50 functional groups of 3 main ontologies: the biological process was the largest class followed by cellular components and molecular function class (Figure 3). Based on the GO, we found that the majority of genes were enriched for the three largest terms, such as oxidation-reduction process, chitin-based cuticle development and proteolysis. The main DEGs of GO terms were mainly enriched for cellular components class, such as extracellular space, cytoplasm and extracellular space matrix. In the category of molecular function, the three subcategories of DEGs enriched for GO terms “structural constituent of cuticle”, “oxide of reductase activity” and “structural constituent of chitin-based larval cuticle” were the most enriched terms (Figure 3).

### 3.6. KEGG Pathway Analysis

**Figure 4:**
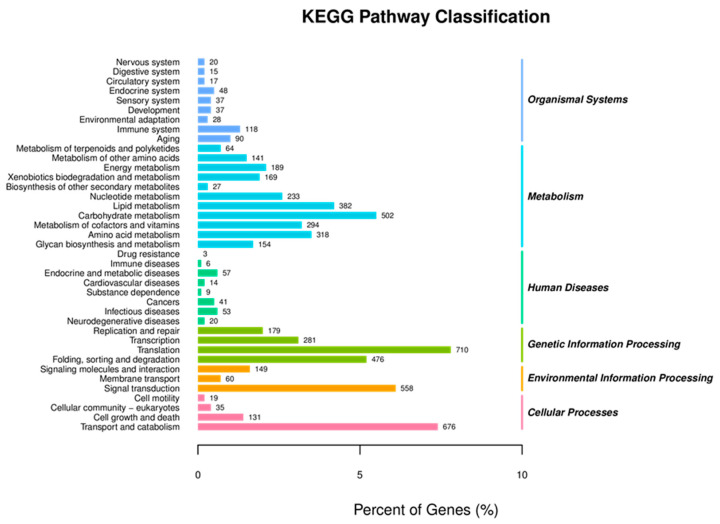
Kyoto Encyclopedia of Genes and Genomes (KEGG) annotation and pathways of the *Spodoptera frugiperda* transcriptome.

Subsequently, the KEGG pathway assignment was also performed on unigenes to identify the biological pathways, including metabolic and regulatory pathways, that will be actively involved in the *S. frugiperda* larvae. The results of KEGG pathways enrichment showed that there were five major KEGG pathways categories, in which Metabolism was the most dominant category of the KEGG pathway (2486), followed by Genetic Information Processing (1646), Cellular Processes (861), Environmental Information Processing (767) and Organismal Systems (410), that are actively involved in different functions (Figure 4). The genes involved in the major subcategories of KEGG pathways included: carbohydrates metabolism (502), xenobiotics biodegradation and metabolism (169), translation (710), folding, sorting and degradation (476), signal transduction (558), transport and catabolism (676) and cell growth and death (131) (Figure 4). This result indicated that in *S. frugiperda* larvae, the three main categories of biological process, cellular component and molecular function were all somehow affected by Noposion Yihaogong^®^ 5% EC insecticide exposure. In the present study, the KEGG pathway assignment will be helpful to further research specific biological processes, functions and pathways that exist in the *S. frugiperda* larvae. The most significant KEGG pathway in the DEGs analysis (KEGG) pathways of *S. frugiperda* after exposure to a Noposion Yihaogong^®^ 5% EC insecticide are shown in Supplementary Figure S1.

### 3.7. Identification of Key Genes and Pathways Involved in Detoxification

**Figure 5:**
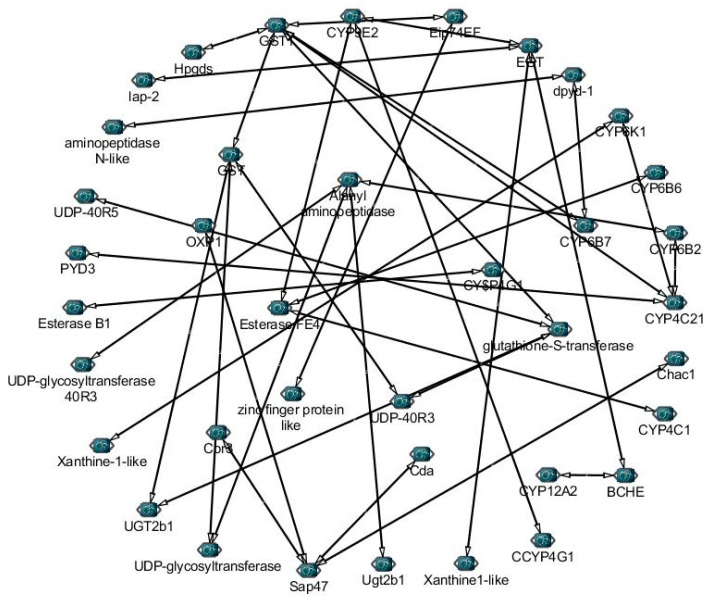
The correlation networks of 52 detoxification genes from different families involved in pathways under Noposion Yihaogong^®^ 5% EC insecticide stress treatment were established based on the Pearson correlation coefficients of these gene pairs using RNA-Seq data. The PCC of co-relation gene pairs was considered significant (*p* < 0.05).

In this study, GO/KEGG enrichment analyses were used to identify metabolic enzymes and related genes involved in detoxification of insecticides. We found several upregulated genes harboring important detoxification enzymes. In recent studies, a number of detoxification genes have been identified from the de novo transcriptome analysis of *S. frugiperda* larvae, with the majority of these genes belonging to the important class of detoxification enzymes, cytochrome P450 (P450s). Cytochrome P450s are an extensively distributed protein superfamily that play a key part in the metabolism and detoxification of a wide range of plant secondary metabolites and synthetic chemicals. Cytochrome P450 enzymes have been divided into four different clades, including CYP2, CYP3, CYP4 and mitochondria. In the present study, upregulated detoxification genes have been identified to include twelve genes relating to cytochrome P450s, three genes from esterase and two genes from UDP-glucuronosyltransferases (UGT) (Figure 5).

### 3.8. Study of Gene Interaction Network with Relation to Detoxification Pathways

**Figure 6:**
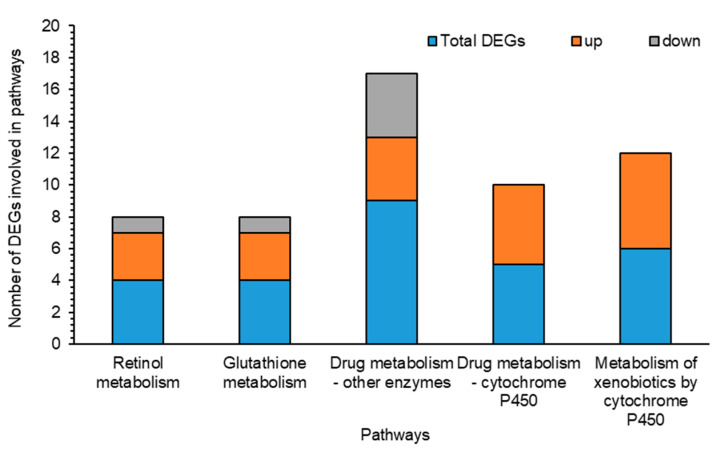
Comparison of up- and down-regulated genes of differentially expressed genes (DEGs) involved in different pathways of *Spodoptera frugiperda* after exposure of its larvae to a sublethal concentration of Noposion Yihaogong^®^ 5% EC insecticide versus the unselected group (CK). The *x*-axis lists the major detoxification pathways’ terms under categories of retinol metabolism, glutathione metabolism and drug metabolism by other enzymes, drug metabolism by cytochrome P450 and metabolism of xenobiotics by cytochrome P450. The *y*-axis is the number of DEGs involved in each pathway under Noposion Yihaogong^®^ 5% EC insecticide stress.

To further understand the roles of detoxification genes, we identified 52 genes from transcriptome under Noposion Yihaogong^®^ 5% EC insecticide stress and analyzed the correlation network based on the Pearson correlation coefficients (PCCs) (Figure 5). Results showed the 33 positive and 32 negative correlations of selected genes involved in detoxification pathways under Noposion Yihaogong^®^ 5% EC insecticide stress. Taking all together, study of the co-expression network show that detoxification genes are greatly influenced under insecticide stress (Figure 5). Furthermore, our results show the important role of major detoxification DEGs involved in five different pathways from transcriptome under Noposion Yihaogong^®^ 5% EC insecticide stress (Figure 6).

### 3.9. Validation of Differentially Expressed Genes (DEGs) Using RT-qPCR

**Figure 7:**
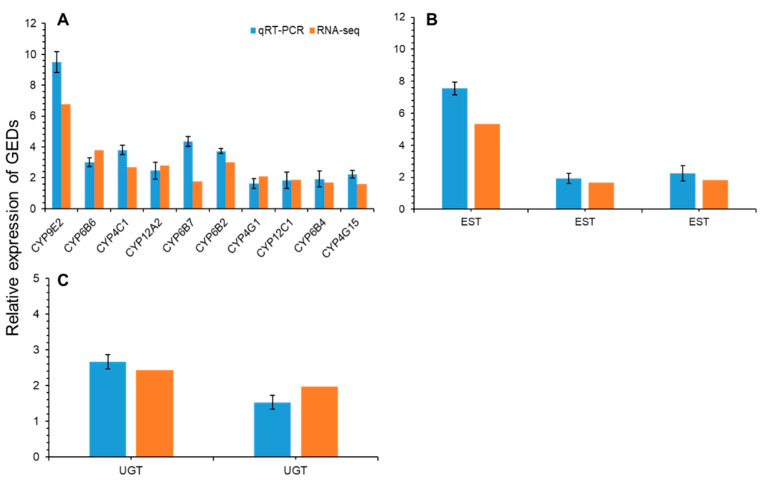
Results for the qRT-PCR confirmation of the DEGs library. (**A**) A qRT-PCR analysis of ten upregulated genes of detoxification genes: P450 (cytochrome P450), (**B**) three upregulated EST (esterases) and (**C**) two upregulated Noposion Yihaogong^®^ 5% EC insecticide-related detoxification genes, UDP-glucuronosyltransferases (UGT). Bars are means ± SE.

To further explore the expression level of the identified detoxification enzyme genes after the treatment of *S. frugiperda* larvae with Noposion Yihaogong^®^ 5% EC insecticide, RT-qPCR analysis was performed to validate the DEGs databases acquired from RNA-sequencing analysis. Twelve DEGs in cytochrome P450s were significantly upregulated in the whole body of Noposion Yihaogong^®^ 5% EC insecticide-treated larvae (Figure 7). This result showed that these detoxification enzyme genes, especially from cytochrome P450, were potentially involved in the detoxification of Noposion Yihaogong^®^ 5% EC insecticide. 

## 4. Discussion

The use of synthetic insecticides is currently the most common method to control fall armyworm in agricultural crops; however, this is only partially effective due to the development of resistance among field populations [16,37]. The rapid evolution of insecticide resistance of many agricultural pests is attributed to various resistance mechanisms and needs attention. Amplification, overexpression and/or modification of genes that encode a number of the microsomal oxidase such as CYPs, GSTs and CarEs groups have been reported to be responsible for metabolic resistance in a range of insect species to carbamates, pyrethroids and organophosphates insecticides [20,50]. Comprehensive biochemical studies showed that various detoxifying enzymes, such as cytochrome P450s (CYPs), glutathione S-transferases (GSTs) and carboxylesterases (CarEe), are induced by the various groups of insecticides in the resistant field strains of the fall armyworm [14]. Furthermore, it has been frequently reported that pyrethroid resistance of major crops’ insect pests is commonly associated with the overexpression of one or more cytochrome P450 genes [51,52].

In this study, the field population of *S. frugiperda* showed greater resistance (31.1-fold) to Noposion Yihaogong^®^ 5% EC insecticide than the lab-susceptible population. Similar to our results, previous studies reported high levels of resistance against pyrethroid insecticides, including lambda-cyhalothrin and fenevalerate (with highest LC_50_ (253.88 and 3000 mg/L) values), from field populations of *S. frugiperda* in different provinces of China [24,53]. However, conventional synthetic pyrethroids may no longer be effective against *S. frugiperda* due to developed resistance. Our results (lambda-cyhalothrin; RR_50_ = 31.2-fold) are consistent with previous studies which also showed that *S. frugiperda* populations in the Americas have developed resistance to pyrethroid insecticides, e.g., permethrin (RR_50_ = 220-fold), zeta-cypermethrin (RR_50_ = 62-fold) and fluvalinate (RR_50_ = 216.1-fold), respectively [6,14,18,54,55]. Furthermore, the transcriptomes of *S. frugiperda* have been generated using short-read sequencing generated by Illumina from various developmental stages exposed to different kinds of synthetic and biopesticides [33,37,56]. Based on the transcription data, the differential expression of unigenes suggests that enriched GOs, and many biological pathways, are associated with specific stages of development and insecticide resistance mechanisms. We analyzed the whole-body transcriptional response of *S. frugiperda* larvae exposed to Noposion Yihaogong^®^ 5% EC insecticide to investigate the differentially expressed detoxification genes and related metabolic pathways. A total of 1819 DEGs were detected between the Noposion Yihaogong^®^ 5% EC insecticide-treated and the unselected group. Of these, 863 (47.44%) DEGs were upregulated and 956 (52.56%) DEGs were shown to be downregulated at significant levels in the Noposion Yihaogong^®^ 5% EC insecticide-treated group as compared with the unselected group. Most of the upregulated genes belonged to the cytochrome P450 superfamily. These results coincide with those documented for *S. frugiperda* Sf9 cells and diamondback moth, *Plutella xylostella,* when treated with lufenuron, harmine and chlorantraniliprole [33,37,57]. In the present study, ten cytochrome P450 genes were commonly upregulated in all treated larvae. Transcription of six CYP450 genes: CYP9E2, CYP6B6, CYP4C1, CYP12A2, CYP6B7 and CYP6B2, increased significantly with Log2FC values of 3.0–6.3. Furthermore, transcription of four of the ten CYP450 genes increased significantly with Log2FC values of 1.5–2 (Figure 7). In previous studies, a similar trend has been documented in other insects, including *S. frugiperda*, in which overexpression of numerous cytochrome P450 genes was observed following exposure to a range of different synthetic insecticides, including non-pyrethroid classes [37,58,59,60]. These cytochrome P450 genes have been reported to play a key role in the metabolism and detoxification with broad substrate specificity that can respond to different insecticides [60,61,62]. Cytochrome P450s have a variety of metabolic functions, with their overexpression acting as the most common insecticide detoxification mechanism in insects [63,64,65]. Therefore, although our results revealed that overexpression of specific cytochrome P450s genes is associated with the exposure to Noposion Yihaogong^®^ 5% EC insecticide in *S. frugiperda*, whether these P450s can actively metabolize Noposion Yihaogong^®^ 5% EC insecticide requires further investigation.

In the transcriptome, we identified five esterase (three up- and two down-regulated) and four UGT-glucosyltransferase unigenes (two up- and two down-regulated) that were significantly affected by Noposion Yihaogong^®^ 5% EC insecticide treatment in the *S. frugiperda* larvae. However, no differential expression of GST genes was observed. This indicates that it may be differential expression of the EST genes which is regulated by the insect’s exposure to insecticides and corresponds to the target insect’s tolerance or susceptibility [66]. The upregulation of UGT genes in response to insecticide exposure has been observed in different insecticide systems [59,67]. Interestingly, previous studies have also described downregulation in the transcription level of two UGTs in the antennae of a female *Spodoptera littoralis* following exposure to semiochemicals [68], demonstrating their varied roles in insects. Our findings indicate that cytochrome P450, UGT and EST genes together play an important role for *S. frugiperda* larvae in mediating insecticide-induced stress and in the detoxification of Noposion Yihaogong^®^ 5% EC insecticide.

In addition to detoxification, reduced sensitivity and permeability are important at the target site for tolerance to insecticides. In the present investigation, transcription of all the cuticle protein genes were commonly differentially downregulated in the treated *S. frugiperda* larvae. Cuticle proteins are thought to be the first barrier used by insects to combat the penetration of xenobiotics and regulate water loss. Contrary to our results, several studies have reported differential expressions of cuticular genes in insects following exposure to insecticides [35,59,69,70]. This is identified as a protective mechanism by which the thickening of the cuticle will reduce penetration of the insecticides. However, present findings suggest that downregulation of cuticular genes may contribute to decreasing tolerance in *S. frugiperda* larvae to Noposion Yihaogong^®^ 5% EC insecticide, which eventually leads to their increased susceptibility to stress induced by insecticides. Furthermore, we identified downregulated transcription of three nose resistance fluoxetine genes (NRF6) in Noposion Yihaogong^®^ 5% EC insecticide-treated larvae. In previous studies, NRF6 has been shown to be involved in the acquisition and transportation of various molecules, including several xenobiotics compounds that exhibited increased expression against xenobiotic-induced stress in various insects [35,71]. In addition, one nuclear hormone receptor (FTZ-F1) gene also exhibited downregulated expression following the insecticide treatment. These nuclear hormone receptors can be used as transcription activators to initiate the expression of particular metabolic enzymes (cytochrome P450s), which promote detoxification [72]. In addition, many annotated DEGs were dynamically involved in key detoxification KEGG pathways, including retinol metabolism, glutathione metabolism, drug metabolism by other enzymes, drug metabolism by cytochrome P450 and metabolism of xenobiotics by cytochrome P450 (Figure 6). This demonstrates their association with mitigating the adverse effects of insecticide exposure for larvae. Many xenobiotic metabolites are lipophilic, making them difficult to excrete from the body. As a result, these lipophilic substances accumulate and are often antagonistic to biological processes in the organism [73]. Our transcriptomic results suggest that *S. frugiperda* larval tolerance to insecticides does not take place by the regulation of a single gene, but rather as a result of multiparty regulation by multiple detoxification genes.

In conclusion, the molecular functions of individual *S. frugiperda* genes and the related signal transduction and metabolic pathways remain largely unclear. Our present results show that Noposion Yihaogong^®^ 5% EC insecticide can induce a significant change in the whole-body transcriptomic profile of *S. frugiperda* larvae. The upregulated expression of several detoxification-related genes, GO terms and KEGG pathways may be important to pesticide detoxification in *S. frugiperda*. Together, this transcriptome will serve as a valuable community resource for studies investigating the molecular underpinnings of detoxification genes in fall armyworm and provide a reference for the development of effective strategies to control fall armyworm.

## Figures and Tables

**Table 1:** Toxicity of Noposion Yihaogong^®^ 5% EC insecticide to early third-instar *Spodoptera frugiperda* larvae.

Treatments	n ^a^	(µg-g^−1^) (95% FL) ^b^		Slope ± SE ^c^	χ^2^ ^d^	df ^e^	RR ^f^
		LC_50_	LC_25_				
Susceptible	630	1.3 (1.05–1.52)	0.54 (0.38–0.71)	1.9 ± 0.19	0.99	4	---
Field-population	630	40.6 (33.3–48.9)	15.9 (10.5–20.4)	1.8 ± 0.17	1.8	4	31.2

^a^ Number of tested larvae; ^b^ (95% FL) fiducial limits; ^c^ SE: Standard error; ^d^ Chi-square value (χ^2^); ^e^ degrees of freedom (df); ^f^ RR = Resistance ratio: LC_50_ value of insecticides of Noposion Yihaogong^®^ 5% EC insecticide resistance population divided by LC_50_ value of insecticides of susceptible population.

**Table 2:** Summary of the sequencing data.

Samples	Raw Reads	Clean Reads	Raw Bases	Clean Bases	Valid%	Q20%	Q30%	GC%
unselected -1	53,871,434	51,566,052	8.08 G	7.21 G	95.72	97.94	93.68	50.08
unselected -2	54,235,678	51,465,482	8.14 G	7.20 G	94.89	97.98	93.77	50.24
unselected -3	56,970,122	54,229,496	8.55 G	7.58 G	95.19	97.92	93.62	50.29
Noposion Yihaogong^®^ 5% EC-1	55,781,304	53,123,060	8.37 G	7.43 G	95.23	98.02	93.85	49.28
Noposion Yihaogong^®^ 5% EC-2	57,178,320	54,393,760	8.58 G	7.61 G	95.13	97.95	93.68	50.20
Noposion Yihaogong^®^ 5% EC-3	44,957,636	42,904,598	6.74 G	6.00 G	95.43	98.05	93.92	50.30

**Table 3:** Unigenes annotation against six public databases (DB).

DB	Number of Unigenes	Ratio (%)
Nr	13,585	50.66
GO	9115	33.99
KEGG	9105	33.96
Pfam	9434	35.18
Swissport	8055	30.04
eggNOG	12,465	46.49
All databases	26,814	100.00

## Data Availability

The data presented in this study are available in article

## Supplementary Materials

The following are available online at https://www.mdpi.com/2075-4450/12/2/132/s1, Figure S1: The most enriched Kyoto Encyclopedia of Genes and Genomes (KEGG) pathways of Spodoptera frugiperda after exposure to a 5% lambda-cyhalothrin. The pathways’ significance are shown together with their q-value (color), rich factor (vertical ordinate), and a number of involved genes (size of circles). Table S1: Total number of reads and Table S2: Primers used for qRT-PCR.
