# Supplementary material for: De Novo Transcriptomic Analyses Revealed Some Detoxification Genes and Related Pathways Responsive to Noposion Yihaogong® 5% EC (Lambda-Cyhalothrin 5%) Exposure in Spodoptera frugiperda Third-Instar Larvae
Source: Insects. 2021 Feb 3;12(2):132. doi: 10.3390/insects12020132 (PMC7913311; doi:10.3390/insects12020132)
Supplement: Supplementary file 1 [file insects-12-00132-s001.pdf]

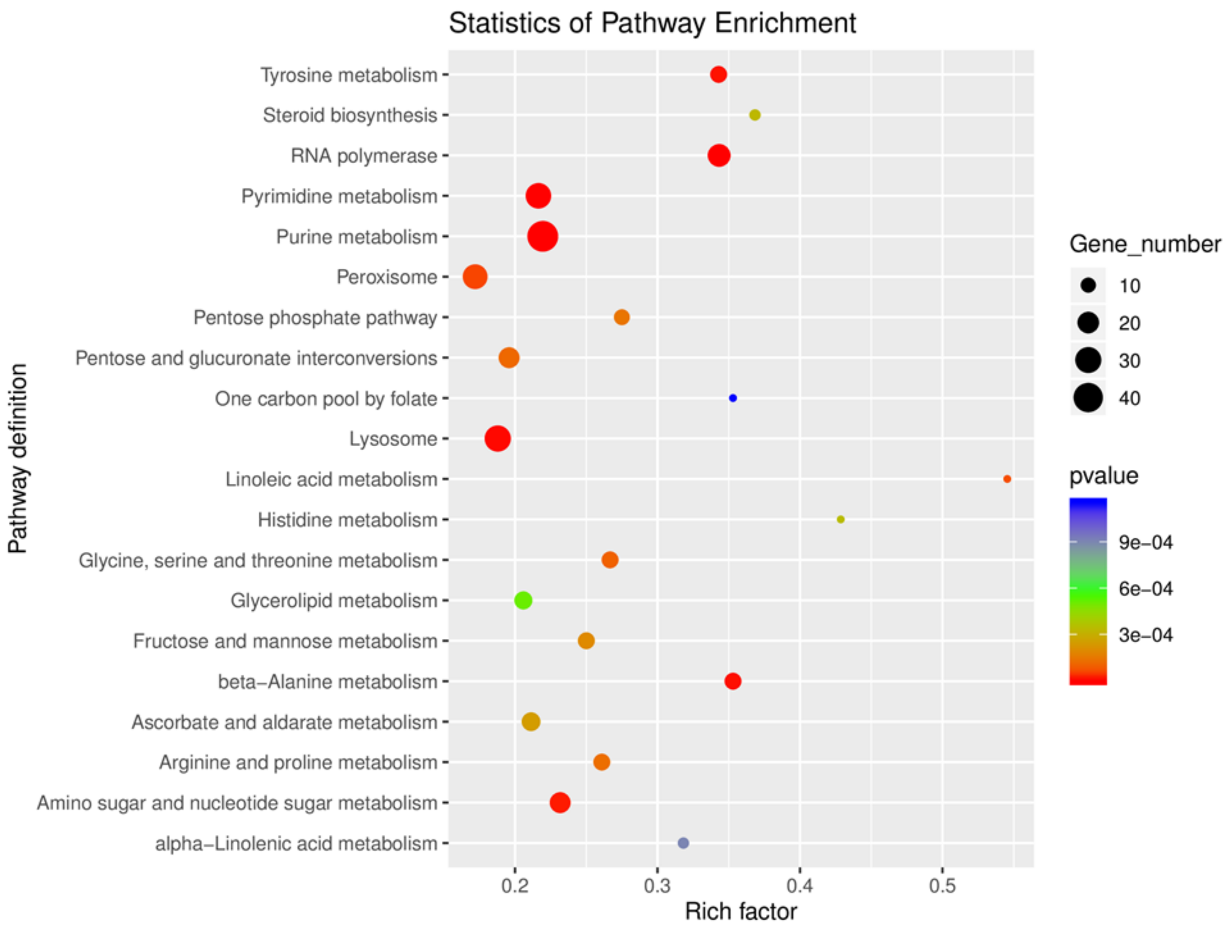

Figure S1. The most enriched Kyoto Encyclopedia of Genes and Genomes (KEGG) pathways of *Spodoptera frugiperda* after exposure to a lambda-cyhalothrin. The pathways' significance are shown together with their q-value (color), rich factor (vertical ordinate), and a number of involved genes (size of circles).

Table S1. Primers used for qRT-PCR.

Transcriptome ID	Gene Name	Primer Name	Sequence(5'-3')
TRINITY_DN16145_c0_g4	CYP9E2	CYP9E2-F	CACACTGAGTCCAGCGTTCA
		CYP9E2-R	TGTAGCGACCGCATCATCTG
TRINITY_DN14149_c0_g1	CYP6B6	CYP6B6-F	TGAGCCTTTGATGCGGAACT
		CYP6B6-R	TCAGCTTGCCACTCGTGAAT
TRINITY_DN13506_c0_g1	CYP4C1	CYP4C1-F	GCAGGAATCACCGAGGAAGT
		CYP4C1-R	TGAGGTTCGTTCGCAAGTCT
TRINITY_DN17120_c0_g1	CYP12A2	CYP12A2-F	GTACTGGCGAAATTGTTGGC
		CYP12A2-R	GCCAGGAGGTAAAGTGTAGC
TRINITY_DN14412_c0_g1	CYP6B7	CYP6B7-F	GGCTTACAGCTCCACGGATT
		CYP6B7-R	GCTGAGGTATCAGTACCGGC
TRINITY_DN15260_c0_g2	CYP6B2	CYP6B2-F	GTAGAACTTGCGCCGGGTAT
		CYP6B2-R	CTGCCAACCCTCTCGTTCAA
TRINITY_DN16042_c0_g2	CYP4G1	CYP 4G1-F	TGCCTTTTAGCCATGGACCC
		CYP 4G1-R	CGACATCAGATGGTGGGAGA
TRINITY_DN13863_c2_g1	CYP12C1	CYP 12C1-F	ATCAACCCTTTGGTTTCGGC
		CYP 12C1-R	GGGCCGAACCATTCCACTT
TRINITY_DN17097_c0_g1	CYP6B4	CYP6B4-F	GATGCTGATTGCGCAGAGTG
		CYP6B4-R	TGTATCTCTGGGTGCTTCGC
TRINITY_DN11687_c1_g7	CYP 4G15	CYP4G15-F	TGACTGAAGACCAGTTCGCC

		CYP4G15-R	TAAGCGACGTTGGGGATTGG
TRINITY_DN15051_c0_g1	Esterase-FE4	ESt-FE4-F	CTTCGGACAATCAGGAGTCA
		ESt-FE4-R	GTTGGTATCGATGGACCAGT
TRINITY_DN16166_c0_g1	Esterase-6	ESt-6-F	CCTCGAAGGCTATCATCGAC
		ESt-6-R	CACAGCAACACTGGATCGTA
TRINITY_DN9351_c0_g1	Esterase-B1	ESt-B1-F	TCTCAACGTGTACACACCAA
		ESt-B1-R	GTATAGGTCGTCGTCACCAG
TRINITY_DN15875_c1_g2	UGT-2B15	UGT-2B15-F	GCTTCGGTACAAACGTGATT
		UGT-2B15-R	GTAGTTCGTCATCGTCCCAT
TRINITY_DN14554_c0_g1	UGT-33B15	UGT-33B15-F	GCTGATGACGGAAGCTTGTA
		UGT-33B15-R	CGATCCCATCGTTTCCAAGT
GenBank: KC262638.1	GAPDH	GAPDH-F	CGGTGTCTTCACAACCACAG
		GAPDH-R	TTGACACCAACGACGAACAT
NCBI locus AF400225	S30	S30-F	CACCCTCGGTGTTAGACGTT
		S30-R	CCACCGGGAAAGTGATACTGT

Table S2. Total number of reads.

Index	All	GC%	Min Length	Median Length	Max Length	Total Assembled Bases	N50
Transcript	66501	40.87	201	577	12042	58723120	1333
Gene	26814	40.78	201	516.00	12042	24365671	1549
